# That Cancer Case

**Published:** 1885-07-20

**Authors:** 


### THAT CANCER CASE.

It is true that a case of unquestionable cancerous disease, seated upon the side of the neck, and involving the parotid gland—a case of long standing. and well advanced, having been once removed by the knife and returned, and presenting a most malignant and unfavorable appearance some four months ago, when its present treatment was commenced—is *now being actually cured* in the Hospital of the SOUTHERN MEDICAL COLLEGE in Atlanta. The case has been in charge of Professor Gaston, and the remedy, Arsenic, as suggested by Dr. Lawson, of Georgia, and set forth in a paper published in the SOUTHERN MEDICAL RECORD of December, 1884. A full report of the case will be published in this journal as soon as the case is complete. So marked and so wonderful is the effect of the remedy upon the disease, and so uniform and rapid has been the improvement in the case, that no doubt is now entertained of a positive and permanent cure.

